# Supplementary figures and images for: Comparing endoscopic mucosal resection with endoscopic submucosal dissection in colorectal adenoma and tumors: Meta-analysis and system review
Source: PLoS One. 2023 Sep 28;18(9):e0291916. doi: 10.1371/journal.pone.0291916 (PMC10538725; doi:10.1371/journal.pone.0291916)

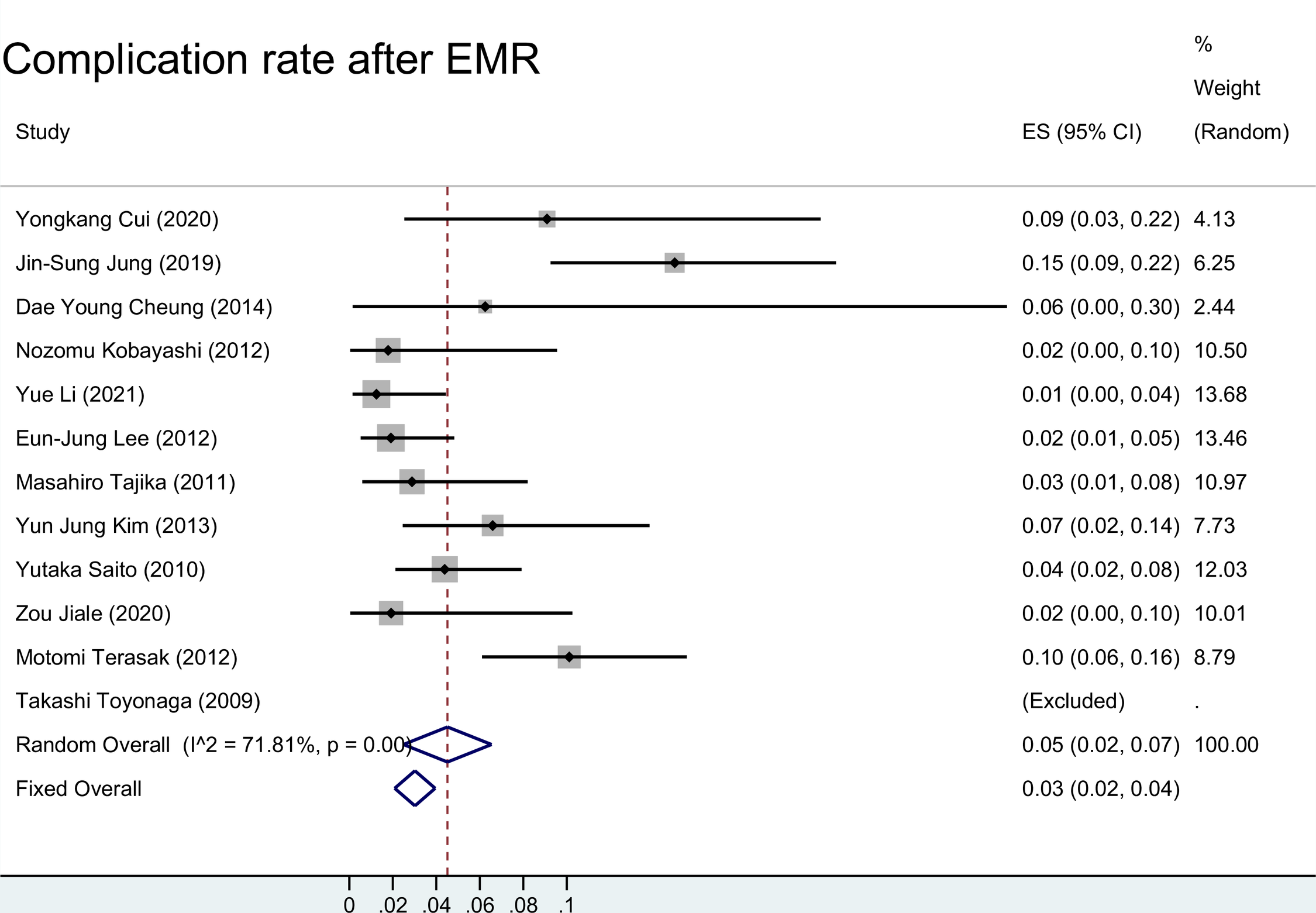

Supplement: S1 Fig — a. Complication rate after EMR. b. Complication rate after ESD. (ZIP) [file pone.0291916.s001.zip › S1a Fig.tif]

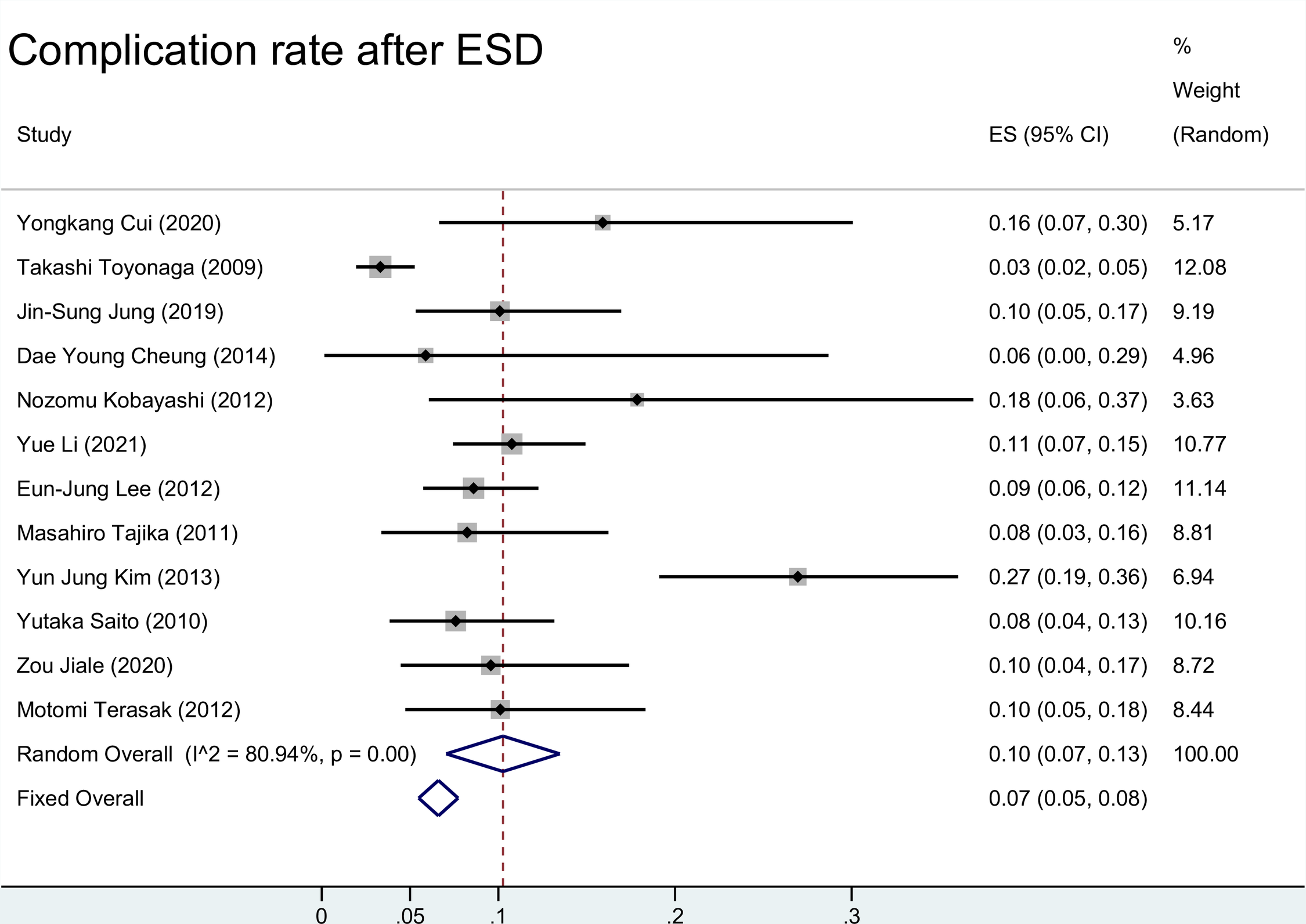

Supplement: S1 Fig — a. Complication rate after EMR. b. Complication rate after ESD. (ZIP) [file pone.0291916.s001.zip › S1b Fig.tif]

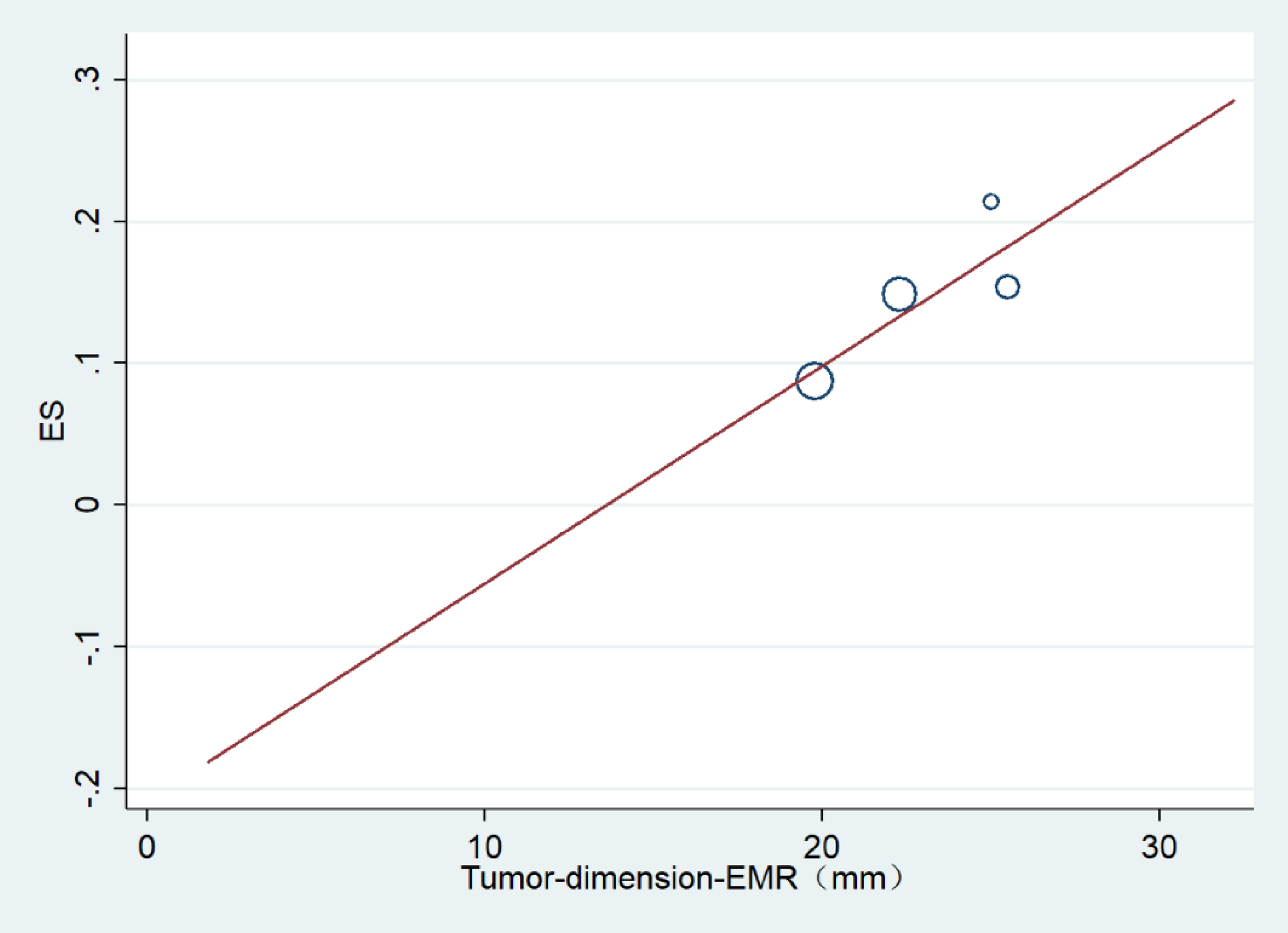

Supplement: S2 Fig — a. Meta-regression analysis for recurrence rate of EMR, tumor size as co-variate. b. Meta-regression analysis for recurrence rate of ESD, tumor size as co-variate. (ZIP) [file pone.0291916.s002.zip › S2a Fig.tif]

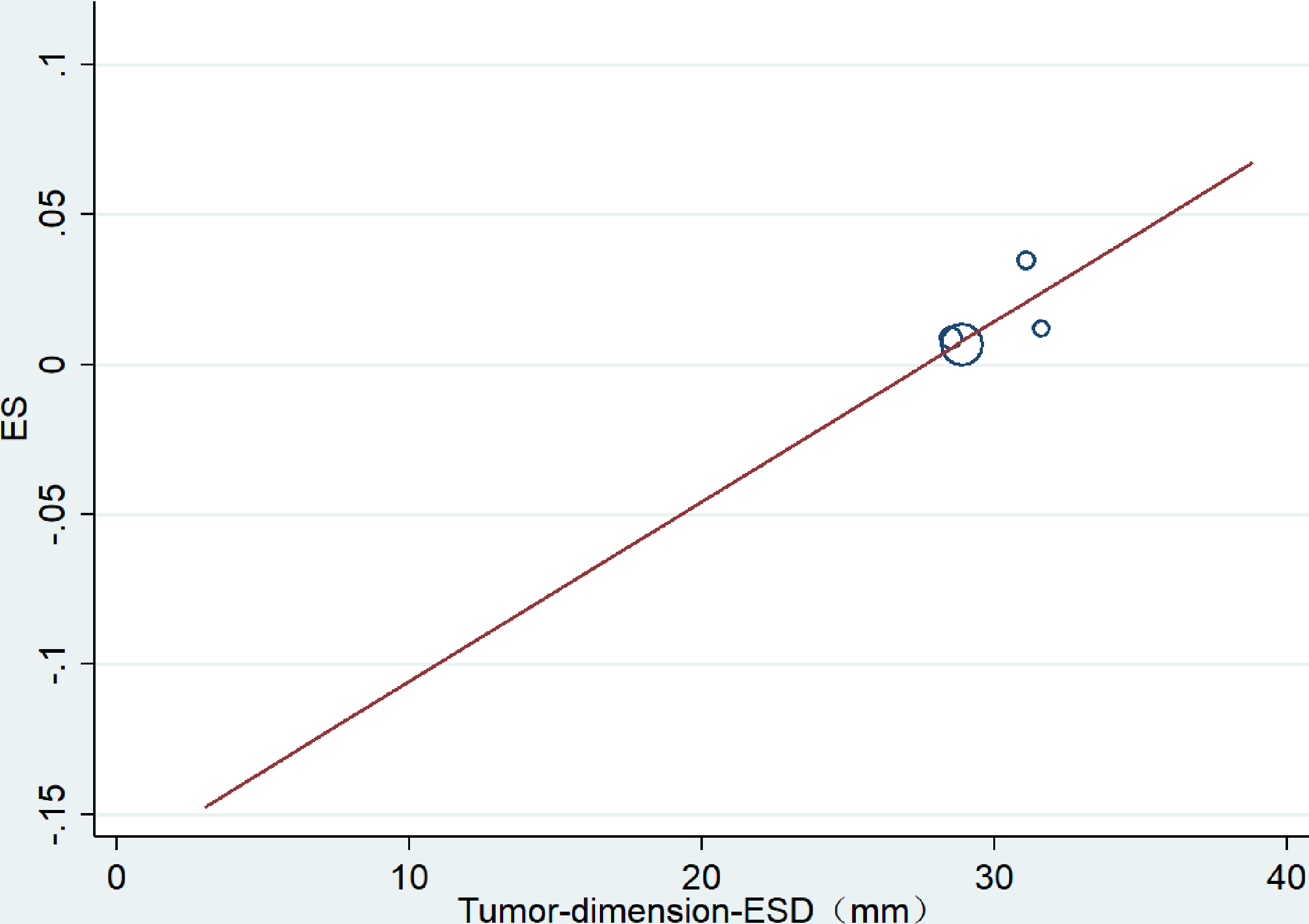

Supplement: S2 Fig — a. Meta-regression analysis for recurrence rate of EMR, tumor size as co-variate. b. Meta-regression analysis for recurrence rate of ESD, tumor size as co-variate. (ZIP) [file pone.0291916.s002.zip › S2b Fig.tif]
