# Supplementary material for: Comparing endoscopic mucosal resection with endoscopic submucosal dissection in colorectal adenoma and tumors: Meta-analysis and system review
Source: PLoS One. 2023 Sep 28;18(9):e0291916. doi: 10.1371/journal.pone.0291916 (PMC10538725; doi:10.1371/journal.pone.0291916)
Supplement: S1 Table — (DOCX) [file pone.0291916.s003.docx]

**Supplementary table 1:** Result of meta regression analyses (Trowman method) to include tumor size as a co-variate and surgery time (EMR vs ESD) as the dependent variable.

| **fmean** | **Coef.** | **Robust Std. Err.** | **t** | **P>\|t\|** | **95% Conf. Interval** | |
| --- | --- | --- | --- | --- | --- | --- |
| **treat** | -37.64 | 7.76 | -4.85 | 0.001 | -55.52 | -19.76 |
| **bmean** | 1.34 | 0.30 | 4.53 | 0.002 | 0.66 | 2.03 |
| **_cons** | 34.60 | 9.11 | 3.80 | 0.005 | 13.58 | 55.61 |
